# Enhanced acquired antibodies to a chimeric *Plasmodium falciparum* antigen; UB05‐09 is associated with protective immunity against malaria

**DOI:** 10.1111/pim.12445

**Published:** 2017-06-22

**Authors:** J. N. Dinga, S. D. Gamua, V. P. K. Titanji

**Affiliations:** ^1^ Faculty of Science Biotechnology Unit University of Buea Buea South West Region Cameroon; ^2^ Cameroon Christian University Institute Bali Cameroon Cameroon

**Keywords:** antibody response, ELISA, immune protection, malaria, UB05‐09 chimera

## Abstract

It has been shown that covalently linking two antigens could enhance the immunogenicity of the chimeric construct. To prioritize such a chimera for malaria vaccine development, it is necessary to demonstrate that naturally acquired antibodies against the chimera are associated with protection from malaria. Here, we probe the ability of a chimeric construct of UB05 and UB09 antigens (UB05‐09) to better differentiate between acquired immune protection and susceptibility to malaria. In a cross‐sectional study, recombinant UB05‐09 chimera and the constituent antigens were used to probe for specific antibodies in the plasma from children and adults resident in a malaria‐endemic zone, using the enzyme‐linked immunosorbent assay (ELISA). Anti‐UB05‐09 antibody levels doubled that of its constituent antigens, UB09 and UB05, and this correlated with protection against malaria. The presence of enhanced UB05‐09‐specific antibody correlated with the absence of fever and parasitaemia, which are the main symptoms of malaria infection. The chimera is more effective in detecting and distinguishing acquired protective immunity against malaria than any of its constituents taken alone. Online B‐cell epitope prediction tools confirmed the presence of B‐cell epitopes in the study antigens. UB05‐09 chimera is a marker of protective immunity against malaria that needs to be studied further.

## 1 INTRODUCTION

The use of vaccines has been shown to be an effective tool in curbing infectious diseases.^1^ A widely effective vaccine is yet to be developed for malaria a devastating infectious disease caused by the protozoon, *Plasmodium falciparum*. Although malaria has receded as a result of intensive control campaigns, it killed 438 000 persons most of them children in sub‐Saharan Africa in 2015, thereby underscoring the need to intensify control measures including the development of an effective vaccine against the disease.

Natural immunity against malaria is mediated in part by antibodies directed against the blood stage parasite, as demonstrated by passive transfer experiments of immunoglobulins.^2^ There are likely many target antigens that mediate protective immunity; however, their identification is obscured by the presence of malaria‐specific antibodies that do not necessarily correlate with immune protection. Current efforts to develop an effective vaccine have been faced with biological constrains especially the complexity of the parasite antigenic profile and the existence of parasite immune evasion mechanisms. However, studies have suggested that protective immunity and vaccination are feasible against malaria.^3^, ^4^, ^5^, ^6^ The recently approved RTS,S vaccine also reiterates this hope, although it is only about 30%‐60% effective.^7^ Furthermore, specific serum antibodies correlated with immune protection against malaria after vaccination.^8^ A broadly effective vaccine would thus be a great asset in controlling the disease.^9^

It has also been shown recently that vaccine constructs containing more than one antigen can elicit high humoral and cellular immune responses in human volunteers^10^ and offer protection against malaria in young African children.^11^ The fusion of two antigens to generate a chimera rather than simply mixing two antigens has been shown to enhance the immunogenicity of the constituent antigens.^12^, ^13^ One of the properties of a good malaria vaccine candidate is that the immune response induced should correlate with protective immunity against malaria. This protection involves the absence of malaria which is manifested by the absence of fever and parasitaemia.^14^, ^15^ By contrast, the presence of fever and parasitaemia constitute a significant risk factor for a poor clinical outcome.

Sero‐epidemiological studies have been used to identify most malaria vaccine candidates. In a similar study by our group that employed differential immune‐screening among other techniques, UB05 antigen was also identified, further characterized and proposed as a potential marker of protective immunity against malaria.^15^ UB09 was also identified in the same study and was later shown to induce T‐cell responses that correlated with immune protection from malaria.^16^ Sequence analyses of UB09 show that it is homologous to the circumsporozoite‐related antigen (CRA), which covers the C‐terminal fragment of CRA.^16^ The aims of the present investigation were to determine whether antibody responses to UB09 correlate with protective immunity to malaria and furthermore to find out whether the chimera UB05.09 could be employed as a reagent to delineate between semi‐immune and susceptible malaria subjects.

## 2 MATERIALS AND METHODS

### 2.1 Ethical clearance

Ethical clearance for this study was obtained from the Faculty of Health Sciences‐Institutional Review Board, ref: 2013/144/UB/FHS/IRB.

### 2.2 Study site

The study was carried out in a malaria‐endemic area, Buea, Cameroon. Buea is a multi‐ethnic town with endemic malaria found along the flanks of Mount Cameroon in the South West region of the Republic of Cameroon. Malaria prevalence varies between a hyperendemic (rainy season) and meso‐endemic (dry season) zone with perennial malaria transmission.^17^

### 2.3 Study subjects

A general invitation was given to the people resident in Buea and those who visited the Buea Regional Hospital for medical check‐up in order to recruit subjects for this study. After explaining the objectives of the research to voluntary individuals, informed consent was obtained from each participating subject above the age of 18 or parents/guardians of sick children below the age of 5. The medical history of each subject was recorded by a collaborating State‐Registered Medical Nurse who also assisted in completing a questionnaire form. Blood was then drawn by a Medical Laboratory Technician for the diagnosis of malaria and serology as described below.

The following criteria were used to screen and admit subjects into the study; (i) subjects who were aged 18 years or older and who had resided in the study site for at least 3 years; (ii) subjects who have not had a malaria episode in the last 12 months, no parasitaemia and fever at sample collection, no prophylaxis, no use of mosquito bed net and had been exposed to mosquito bites were designated semi‐immune subjects (SIS); (iii) subjects who had parasitaemia and fever at sample collection and at least one malaria episode in the last 12 months were referred to as frequently sick subjects (FSS); and (iv) children below the age of five who had fever and parasitaemia were called sick children (SC). This cohort of individuals were highly selected and described previously.^15^, ^18^

### 2.4 Limited longitudinal study and sample collection

Volunteers were prescreened for recruitment into any of the above groups by assessing their malaria status. Slides were prepared for staining using blood taken by finger pricking and stained with Giemsa. The slides were read under an oil immersion microscope (Unico Microscope; series: G380) by two Microscopists at 1000× and 100 fields counted.

5 mL of venous blood from adults and 1 mL from children were collected from recruited subjects in Vacutainer tubes containing EDTA and kept at +4°C for 1 hour. Samples were centrifuged and the plasma aliquoted and stored at −20°C until use.

The SIS were followed up continuously for 2‐4 months and tested for the presence of *P. falciparum* parasites in their blood in this limited longitudinal study. The body temperature of the study subjects was also noted.

### 2.5 Study antigens

The study antigens employed for this work were recombinant UB05 (r‐UB05), recombinant UB09 (r‐UB09) and recombinant UB05‐09 (r‐UB05‐09). These antigens were prepared as earlier described.^16^

### 2.6 Determination of antibody responses to r‐UB09, r‐UB05‐09 chimera and r‐UB05 by ELISA

Antibody (total IgG) measurement was carried out as earlier described^19^ with modifications. Microtitre plates were coated with 100 μL per well of optimized concentration of 0.625 μg/mL of r‐UB09 and r‐UB05‐09 in PBS overnight at +4°C. Control wells were coated with r‐UB05 and soluble fraction of crude *E. coli* extract. After incubation with antigen, the plate wells were washed thrice with 200 μL of Wash buffer (PBS‐Tween‐20 (0,05%); followed by blocking with 150 μL per well of 0.2% casein in PBS with 0.05% Tween‐20. The plates were washed and plasma added at 1:150 dilutions in Wash buffer containing 1% skimmed milk. The plate was incubated for 3 hours at room temperature. Human plasma from malaria‐naive individuals (kind gift from Mrs. Philomena Gwanmesia of the Biotechnology Center, University of Yaounde I) were used as negative control plasma as well the Tag only (Fusion partner) antigen was used as a negative control antigen. The r‐UB05 antigen and soluble fraction of crude *E. coli* extract were used as positive controls. After incubation, the plates were washed three times and 100 μL of anti‐rabbit IgG‐HRP conjugate (Sigma) diluted at 1:10 000 in Wash buffer containing 1% skimmed milk. Incubation was done at room temperature for 1 hour. After anti‐rabbit IgG‐HRP conjugate incubation, the plates were washed and 100 μL of substrate added. Optical densities were read at 405 nm on a microplate reader (LabsystemsMultiskan MCC 340, Helsinki, Finland). A subject was considered positive if its OD value was equal to or greater than the mean control OD +2 SD. The experiment was run in duplicates.

### 2.7 Antibody epitope prediction using online resources

In silico prediction of antibody epitopes from UB09, UB05 and UB05‐09 was done using algorithms in IEDB Analysis Resources (www.immuneepitope.org).

### 2.8 Statistical analysis

Two‐sample Kolmogorov–Smirnov test was used to determine the significance in the OD values of the various antigens against the immune status of the study subjects. Spearman's rho was used to determine correlation between data sets. Kruskal–Wallis test was used to assess difference between body temperature, parasitaemia and OD values. All these tests were performed using SPSS software (Version 17.0, Chicago, IL, USA).

## 3 RESULTS

### 3.1 Study subjects

Table 1 summarizes the characteristics of the subjects studied. Plasma samples from forty (40) semi‐immune subjects (SIS), thirty‐four (34) frequently sick subjects (FSS) and twenty‐seven (27) sick children (SC) were analysed in this study.

**Table 1:** Summary of demographical information of study subjects

Immune status	Sex	Median age (years)	Median body temperature (°C)	Median parasitaemia (trophozoite/mm^3^)
Semi‐immune subjects (SIS)	Female	21 (19‐33)	37.1 (36.9‐37.4)	0 (0‐0)
	Male	22 (22‐27)	37.2 (37‐37.5)	0 (0‐0)
Frequently sick subjects (FSS)	Female	29.5 (18‐60)	38.7 (37.9‐38.9)	4761 (742‐29867)
	Male	34.5 (19‐60)	38.45 (36.9‐38.9)	3868 (320‐180000)
Sick children (SC)	Female	3.25 (1.6‐5)	38.6 (37.9‐39)	10698 (800‐198000)
	Male	3 (1.5‐4)	38.3 (38‐38.8)	16000 (2346‐79000)

Sick people (FSS and SC) had higher parasitaemia and body temperature compared to SIS (*P*<.0001). SC had higher parasitaemia than FSS who in turn carry more parasites than SIS (*P*<.0001). FSS were significantly older than SIS (*P*<.0001) and SC (*P*<.0001).

Hence, a total of one hundred and one subjects were admitted and stayed till the end of the study. All semi‐immune subjects (SIS) were negative for malaria parasites at the time of sample collection and had no fever. Also, all of the frequently sick subjects (FSS) and the sick children retained for the study had fever and were positive for malaria parasites upon the examination of Giemsa‐stained thick blood smears, at the time of blood sample collection. Parasitaemia was mostly high and ranged from 742 to 198 000 parasites/mm^3^ (Table 1).

### 3.2 Characterization of recombinant UB09

**Figure 1:**
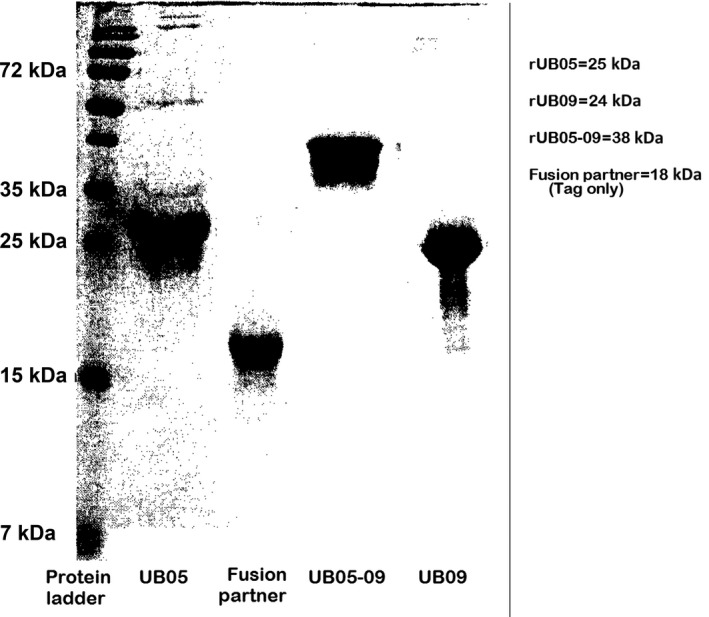
SDS‐PAGE of overexpressed and purified study antigens showing apparent homogeneity

The *UB09* gene as identified in a previous study^15^ is homologous to CRA. The full *CRA* gene is made of three exons and two introns. The r‐UB09 antigen covers part of exon 2 and complete exon 3. In terms of amino acid sequence, it covers the C‐terminal fragment of CRA, that is, r‐UB09 is 100% identical to amino acid residues 89‐162 of CRA. UB09 is therefore a fragment of the full CRA antigen.^16^ As earlier described,^16^ the study antigens were cloned, sequenced and overexpressed, purified to apparent homogeneity and checked with SDS‐PAGE (Figure 1).

### 3.3 Comparison of the immunoglobulin G responses to the antigens UB09, UB05 and their chimera UB05‐09

**Figure 2:**
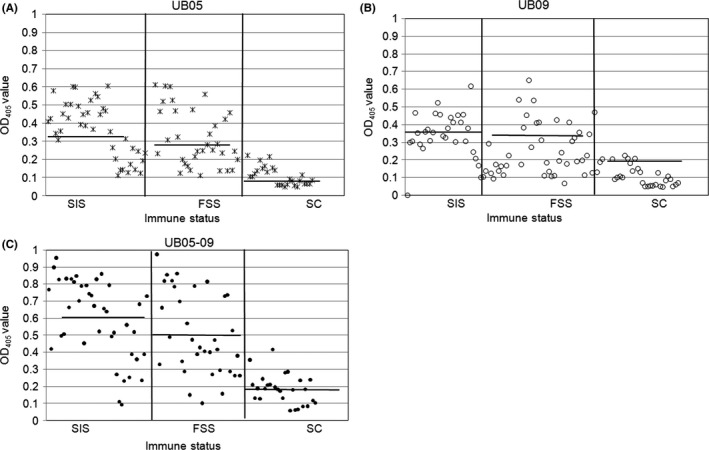
Use of ELISA to detect antigen‐specific antibody in human plasma. The antigens, UB09 (A), UB05 (B) and UB05‐09 (C), were used in ELISA to test for the presence of antibodies in human plasma in a malaria‐endemic area. The experiment was run in duplicates. SIS, semi‐immune subjects; FSS, frequently sick subjects and SC, sick children

Immunoglobulin G responses to r‐UB09 were tested in 101 subjects of different clinical groups namely semi‐immunes, frequently sick and children (whose immunity to malaria is known to be poorly developed). It was observed that that the level of antigen‐specific antibody was far lower in sick children compared to adults (*P<*.0001) (Figure 2A). As shown in Figure 2A, specific to r‐UB09 increased with age. Among adults, it turned out antibody levels to r‐UB09 were averagely higher, but not significantly different in SIS than in FSS (Figure 2A). Immunoglobulin G levels against r‐UB05 followed the same trend as that observed for r‐UB09, in that antibodies to r‐UB05 were lower in sick children compared to adults (*P<*.0001) (Figure 2B). Also, among the adults recruited for the study, the antibody level to r‐UB05 was also higher in SIS than in FSS even though this was not statistically significant (Figure 2B).

**Figure 3:**
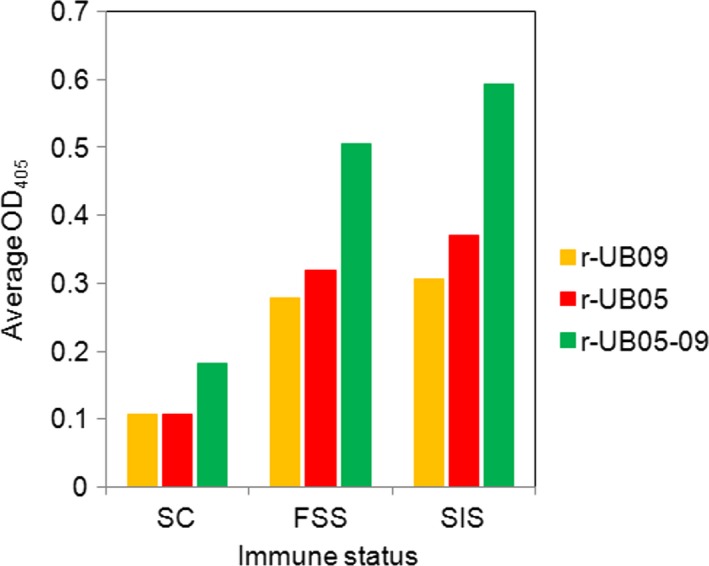
Average OD values shows chimeric UB05‐09 enhances recognition of the antigen by antibodies in human plasma. *P*<.031. SC, sick children, FSS, frequently sick subjects, SIS, semi‐immune subjects

To find out whether fusing UB09 to UB05 to generate a chimera would enhance the diagnostic potential of the components, a chimera termed UB05‐09 was cloned, expressed and used to probe for antibodies in the plasma of the study subjects. Statistical analyses revealed that adults possessed a far higher concentration of antibody to the chimera than sick children (*P<*.0001), and unlike in r‐UB09 and r‐UB05, antibody to the chimera was significantly greater in SIS than in FSS (*P<*.031) (Figure 2C). Comparing the responses showed that the antibody response to the r‐UB05‐09 chimera almost doubled that of r‐UB09 and r‐UB05 alone (Figure 3). Considering the ability of rUB05‐09 to act as a reagent for diagnosing acquisition of some immunity against malaria, the specificity of UB05 was 85% and this was increased to 95% for UB05‐09. The negative predictive value for UB05 was 39.1% and this was increased to 76% when using UB05‐09. Similar trends were observed for the positive predictive value which was enhanced from 57% for UB05 to 84.6% for UB05‐09 (Table 2).

**Table 2:** Comparison of the sensitivity, specificity and positive predictive values of using UB05, UB09 and UB05‐09 antigens as reagents to differentiate between semi‐immune subjects (SIS) and frequently sick subjects (FSS) in a malaria‐endemic region

Test	Sensitivity (%)	Specificity (%)	Positive Predictive Value (%)	Negative Predictive Value (%)
Antigen				
UB05	13.00	85.00	57.00	39.10
UB09	75.70	77.50	75.70	94.00
UB05‐09	18.00	**95.00**	**84.60**	**76.00**

**Figure 4:**
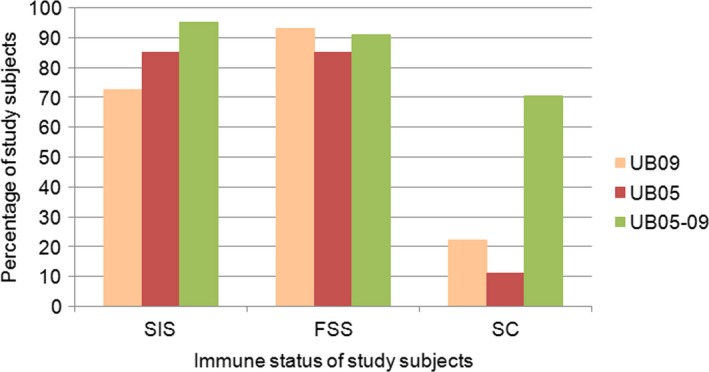
Percentages of study subjects of the different immune status who had antigen‐specific antibody against UB05, UB09 and UB05‐09 chimera. The different subjects in the different immune status group include semi‐immune subjects (SIS), frequently sick subjects (FSS) and sick children (SC). A subject is considered positive if its OD value is equal to or greater than the mean negative control OD+2 standard deviation

In terms of percentage of reactive study subjects, it was observed that 72.5% of SIS, 93% of FSS and 22.2% of SC recognized r‐UB09 (Figure 4). r‐UB05‐09 chimera enhanced the recognition to 94.12% in SIS, 81.4% in FSS and 71.7% in SC. 95% of SIS, 91% of FSS and 70.4% of SC possessed antibody that reacted with the r‐UB05‐09 chimera (Figure 4).

The readouts from the naive plasma wells showed that there was no significant presence of antigen‐specific antibodies in malaria‐naive samples. Also, the fusion partner Tag negative not recognized by plasma from selected study subjects. Any background readings with the fusion partner were deducted from the test.

### 3.4 The effect of febrile status on immunoglobulin G responses to the chimera and its constituent parts

**Figure 5:**
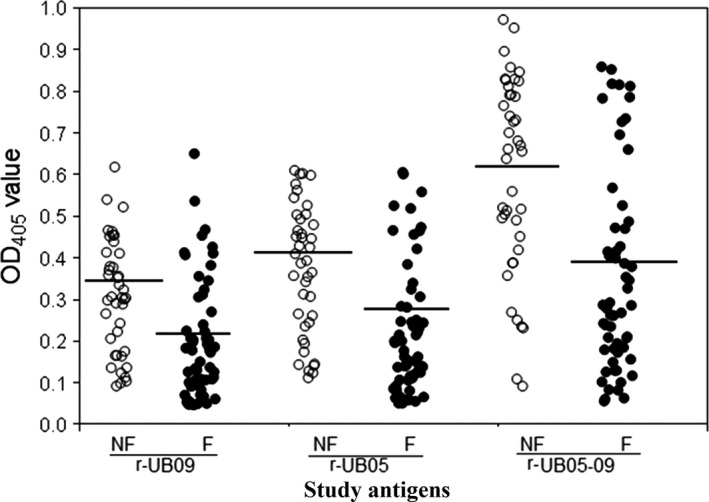
Comparison of fever status of study subjects against their raw OD values measured at 405 nm. OD value is directly proportional to the antigen‐specific antibody level in the human plasma. The antigens tested are UB05, UB09 and UB05‐09 chimera. The OD value of the subjects without fever was significantly higher than those with fever for all antigens tested (*P<*.0001)

To determine the association between fever and antibody response to r‐UB09, a plot was generated showing the febrile status of the study subjects and their respective OD values for antibody response to r‐UB09. Figure 5 indicates that there was a significantly higher level (*P* <.0001) of antigen‐specific antibodies in the plasma of people without fever compared to those with fever for all antigens (r‐UB09, r‐UB05, r‐UB05‐09) studied.

### 3.5 The relationship between antigen‐specific antibody levels and the presence of malaria parasites in peripheral blood

**Figure 6:**
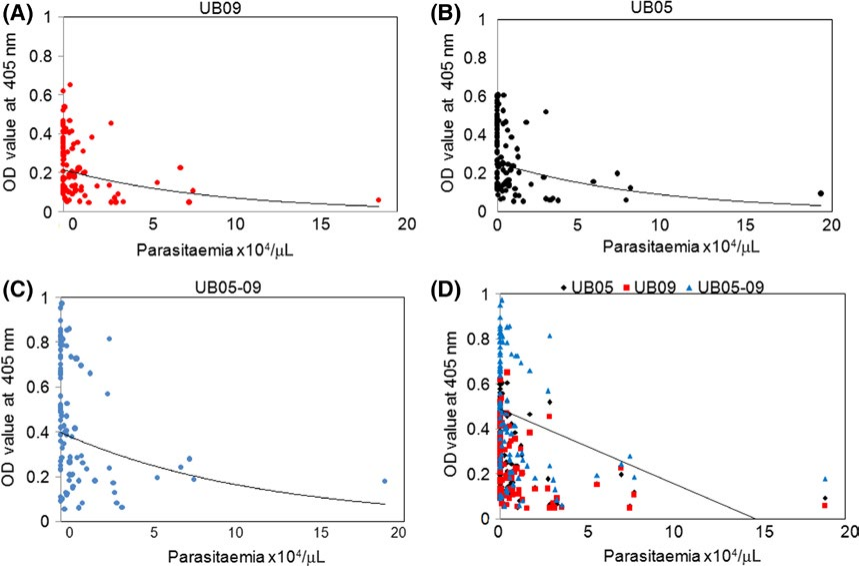
Comparison of antibody response to parasitaemia in human subjects living in a malaria‐endemic area. A: UB09, B: UB05, C: UB05‐09, D: Combined panel of UB09, UB05 and UB05‐09. There is a drop in antibody response as the parasite load in blood increases. This indicates a negative correlation between antibody level and parasitaemia using Pearson correlation. Grouping the study subjects also showed that people with no parasitaemia had a significantly higher level of antigen‐specific antibody compared to those with parasitaemia (*P<*.0001)

To find out whether the level of antibody in plasma correlates with the numbers of malaria parasites in the blood, we compared the OD values obtained to the parasite load of the study subjects. There was a negative correlation between antibody level and parasite load as majority of the subjects with high antibody levels against r‐UB09 had low or no parasitaemia (Figure 6A). When the study subjects were grouped into two groups; “no parasitaemia” and “parasitaemia”, there was a statistically significant difference in antibody levels between the two groups when the measurement was made using r‐UB09 (*P<*.0001). The same trend was observed when the results for r‐UB05 (*P<*.0001) (Figure 6B) and the r‐UB05‐09 chimera (*P<*.0001) (Figure 6C) were analysed. Figure 6D shows the combined panel for r‐UB09, r‐UB05 and r‐UB05‐09 chimera.

### 3.6 There is also a negative correlation between UB05‐09‐specific antibody levels and parasitaemia in sick children

Comparing the antibody levels against the study antigens in sick children showed that children with lower parasitaemia had higher OD values compared to their counterpart with a higher parasitaemia. This was done for sick children who recognized the antigens under study. It was observed that this was true for r‐UB05 and the r‐UB05‐09 chimera and but not with r‐UB09 (data not shown).

### 3.7 UB09 predicted antibody epitopes

**Figure 7:**
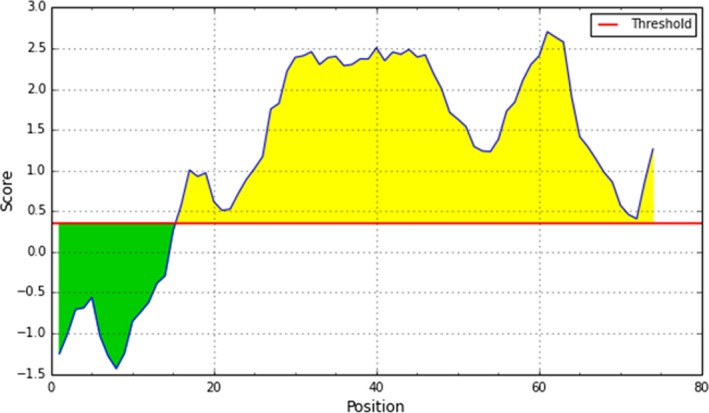
In silico prediction of B‐cell epitopes in UB09 using algorithms found at www.iedb.org

Using Bepipred linear epitope prediction^20^ to analyse the UB09 sequence shows that there are B‐cell epitopes between residues 16 and 74 (Figure 7) with a maximum probability score of 2.703/3. Performing the same in silico analysis using Parker Hydrophilicity Prediction algorithm, B‐cell epitopes are found between residues 16‐18, 26‐47, 52, 55‐67.^21^ Amino acid residue 41 had the highest probability score of 7.329/8 using this algorithm. Kolaskar and Tongaonkar antigenicity prediction method shows antibody epitopes are on amino acid 4‐14, 21‐24, 27, 48‐59, and 70‐71.^22^ There is also a high probability of finding antibody epitopes on residues 14‐20 and 23‐70 as predicted by the Karplus and Schulz flexibility prediction algorithm.^23^ The Emini Surface Accessibility Prediction algorithm indicates that amino acid 14‐20, 28‐44, 56‐64 possess B‐cell epitopes with amino acid 59 having a score of 2.845/3.^24^ Finally, there is a probability of finding B‐cell epitopes on residue 24‐28, 60‐70 according to the Chou and Fasman Beta‐Turn Prediction algorithm.^25^

### 3.8 Online prediction of B‐cell epitopes in UB05

**Figure 8:**
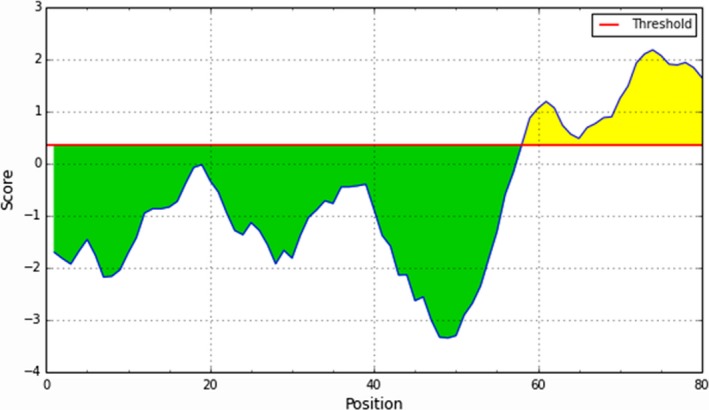
Using computer tools to predict B‐cell epitopes on UB05. Resources at www.iedb.org showed that there is a very strong probability of finding an antibody epitopes between residues 58 and 80 on the UB05 amino acid sequence

To predict the presence of B‐cell epitopes in antigen UB05, we used algorithms in the IEDB Analysis Resources (www.immuneepitope.org). Bepipred Linear Epitope Prediction^20^ shows B‐cell epitopes between residues 58 and 80 (Figure 8). While Parker Hydrophilicity Prediction algorithm predicts B‐cell epitopes between residue 14‐20, 23‐24, 36‐41 and 57‐77.^21^ Also, the Kolaskar & Tongaonkar Antigenicity Prediction method^22^ shows that residues 4‐8, 17‐18, 21‐22, 25‐36 and 40‐67 contain B‐cell epitopes. In silico analysis using the Karplus & Schulz Flexibility Prediction algorithm amino acid 16‐23 and 57‐76 contain antibody epitopes.^23^ When we used the Emini Surface Accessibility Prediction algorithm, we saw that there is a high probability of finding antibody epitopes on residues 15, 16, 18, 20, 23, 35‐37, 40, 57, 59, 61, 62, 65‐77.^24^ Finally, Chou and Fasman Beta‐Turn Prediction algorithm^25^ indicates that residues 15 24 and 56‐77 have high probability of possessing B‐cell epitopes.

### 3.9 B‐cell epitopes found in UB05‐09 chimera using online tools

The chimera UB05‐09 antigen was analysed for the presence of antibody epitopes using the algorithms at IEDB Analysis Resources. The trends found in UB05 and UB09 individual were both present in the UB05‐09 chimera using the different algorithms.

## 4 DISCUSSION

It is now apparent that the development of a multivalent subunit vaccine with widespread application will be a welcomed innovation to tackle the malaria burden. Studies have shown that bringing two antigens into close proximity could enhance their immunogenicity and possibly their protective property and such protection should be associated with the absence of fever and malaria parasite in blood. Here, we study the humoral response of *P. falciparum* antigen; r‐UB09, and the previously studied r‐UB05 and also the chimera of these two antigens called r‐UB05‐09 in the plasma from people living in a malaria‐endemic region to see if the chimera improves the immunogenicity and protective property of the individual component antigens.

In the present study, we generated r‐UB09, r‐UB05 and a chimera thereof, called r‐UB05‐09 and observed that r‐UB09 detected greater levels of antigen‐specific antibodies in the plasma of SIS than in the plasma of FSS and this difference increased using the r‐UB05‐09 chimera. This implies there is an enhancement of the recognition by the immune system of r‐UB05 and r‐UB09 by the r‐UB05‐09 chimera. The bringing of UB05 and UB09 into a single construct could have created an antigen with a different conformation compared to the individual constituent antigens. This could lead to the presentation of more potent epitopes to the immune system, hence the increased recognition of r‐UB05‐09. If UB05‐09 was to be used in a vaccine, then the additive effect would be an advantage as there would be potential for the vaccine to invoke an immune response that target different parts of the parasite.

Earlier it was shown and suggested that UB05 could be a marker of protective immunity against malaria.^15^ This present study also showed that UB05 also induced higher antibody response in SIS than in FSS and far higher when compared with the r‐UB05 response in sick children. This confirms that antibody response to UB05 can be associated with protective immunity against malaria.

An association is not causation, and as such, should not be regarded in isolation. However, when taken with the fever and parasitaemia data, it suggests causation. Given the fact that higher antibody levels, low axial temperature^14^ and little or no parasitaemia^26^ correlate better with protection against malarial infection and disease, it is suggested that r‐UB09 is a marker of protective immunity against malaria and its effect is additive to that of r‐UB05 as shown by the greater efficiency of the chimera r‐UB05‐09 chimeric protein to distinguish between SIS and FSS when compared to its constituents taken individually.

In conclusion, UB09 is a marker of protective immunity to malaria and can be used in combination with another earlier detected antigen UB05 to delineate between semi‐immune and susceptible malaria subjects.

## AUTHOR'S CONTRIBUTION

DJN and VPKT conceived and designed the experiments. DJN and GSD performed the experiments. DJN and VPKT analysed the data. DJN, GSD and VPKT drafted the manuscript or revised it critically for important intellectual content. DJN and VPKT agree to be accountable for all aspects of the work. All authors read and approved the final manuscript.

## DISCLOSURES

None.
